# Supplementary material for: Cellular and molecular associations with intrinsic brain organization
Source: Nat Commun. 2025 Nov 26;16:11641. doi: 10.1038/s41467-025-66291-w (PMC12748960; doi:10.1038/s41467-025-66291-w)
Supplement: Supplementary file 1 — Supplementary Information [file 41467_2025_66291_MOESM1_ESM.pdf]

Supplementary Information for

Cellular and molecular associations with intrinsic brain organization

Guozheng Feng[1], Jiayu Chen[1], Jing Sui[2], Vince D. Calhoun[1*]

[1]Tri-Institutional Center for Translational Research in Neuroimaging and Data Science (TReNDS), Georgia State University, Georgia Institute of Technology, and Emory University, Atlanta, GA, USA;

[2]State Key Laboratory of Cognitive Neuroscience and Learning & IDG/McGovern Institute for Brain Research, Beijing Normal University, Beijing, China.

[*]Corresponding To:

Vince D. Calhoun, PhD, Tri-Institutional Center for Translational Research in Neuroimaging and Data Science (TReNDS), Georgia State University, Georgia Institute of Technology, and Emory University, Atlanta, GA, USA; E-mail: vcalhoun@gatech.edu.

Content:

- **Supplementary Fig. S1. | Spatial maps of ICNs.**
- **Supplementary Fig. S2. | Mitochondrion associations of ICNs.**
- **Supplementary Fig. S3. | Factor analysis for cell-type maps.**
- **Supplementary Fig. S4. | Synergy and relative contributions of distinct neurobiological scales to ICNs.**
- **Supplementary Fig. S5. | Cellular and molecular similarity networks relate to dynamic FNCs at the global level.**
- **Supplementary Fig. S6. | Cellular and molecular similarity networks relate to dynamic FNCs at the regional level.**
- **Supplementary Fig. S7. | Explained variance of diffusion embedding.**
- **Supplementary Fig. S8. | Impact of dimensionality reduction on clustering.**
- **Supplementary Fig. S9. | Cognitive associations of ICNs.**
- **Supplementary Fig. S10. | Cellular and molecular similarity networks relate to cognitive similarity network.**
- **Supplementary Fig. S11. | FNCs relate to cognitive similarity network.**
- **Supplementary Fig. S12. | Dynamic FNCs mediate the connectional associations between cellular and molecular architecture and cognitive function.**

- **Supplementary Fig. S13. | Agreement across cell-type deconvolution methods.**
- **Supplementary Fig. S14. | Consistency of ICN–cell-type associations across thresholds.**
- **Supplementary Fig. S15. | Consistency of ICN–neurotransmitter associations across thresholds.**
- **Supplementary Fig. S16. | Consistency of ICN–mitochondrion associations across thresholds.**
- **Supplementary Fig. S17. | Consistency of cell-type/molecular similarity networks across thresholds.**

Supplementary Figures

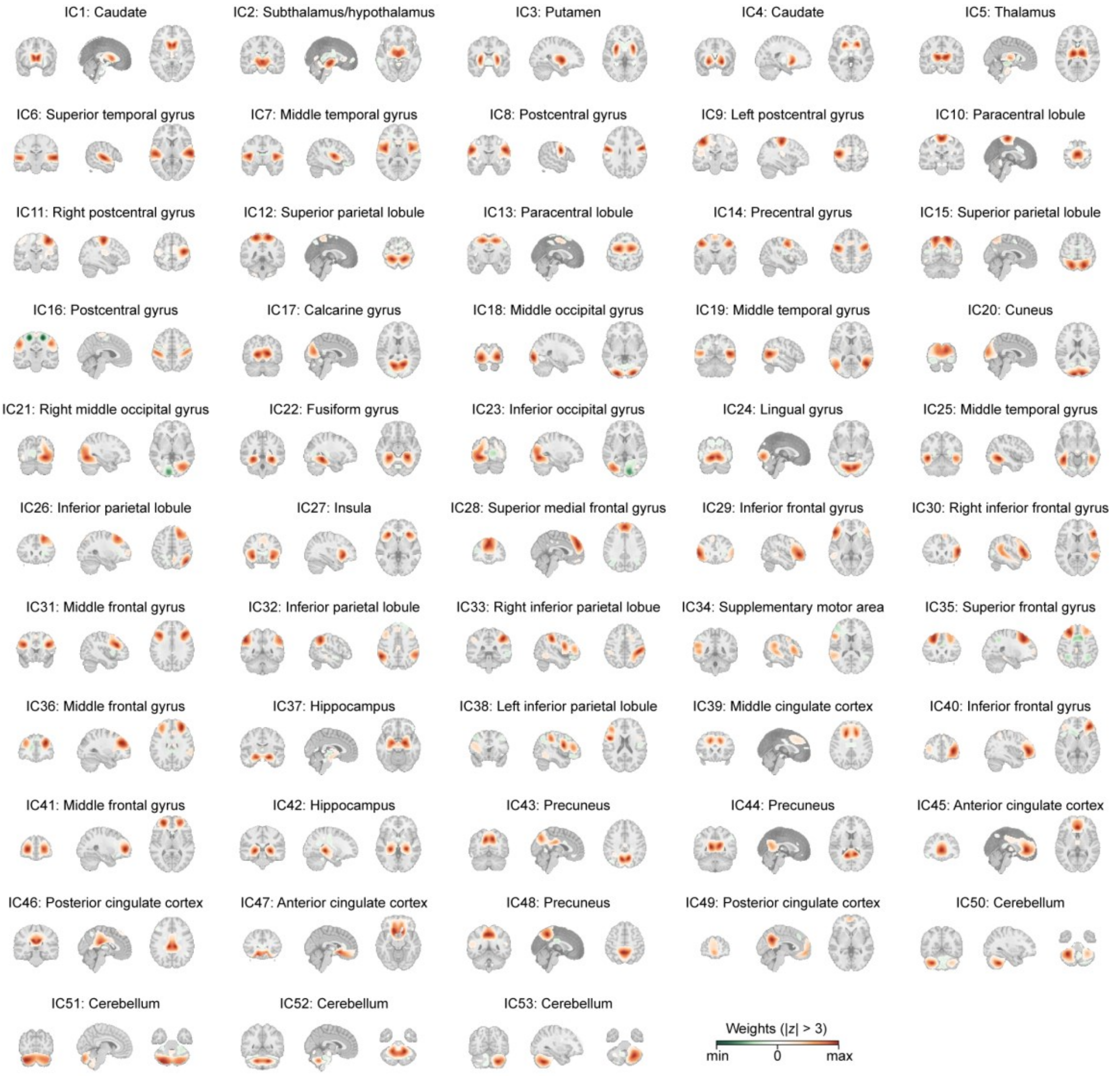

Supplementary Fig. S1. | Spatial maps of 53 ICNs. Fifty-three reproducible and biologically meaningful ICNs were identified using Neuromark_fMRI_1.0[1], derived from rs-fMRI data of 1,828 young adults across the GSP and HCP cohorts. For each ICN, a voxel-wise threshold of |z| > 3 (three standard deviations; a conventional choice to emphasize spatial specificity in ICA maps[2]) was applied to suppress weak activations and emphasize the core or hub regions contributing most strongly to each ICN's spatial pattern.

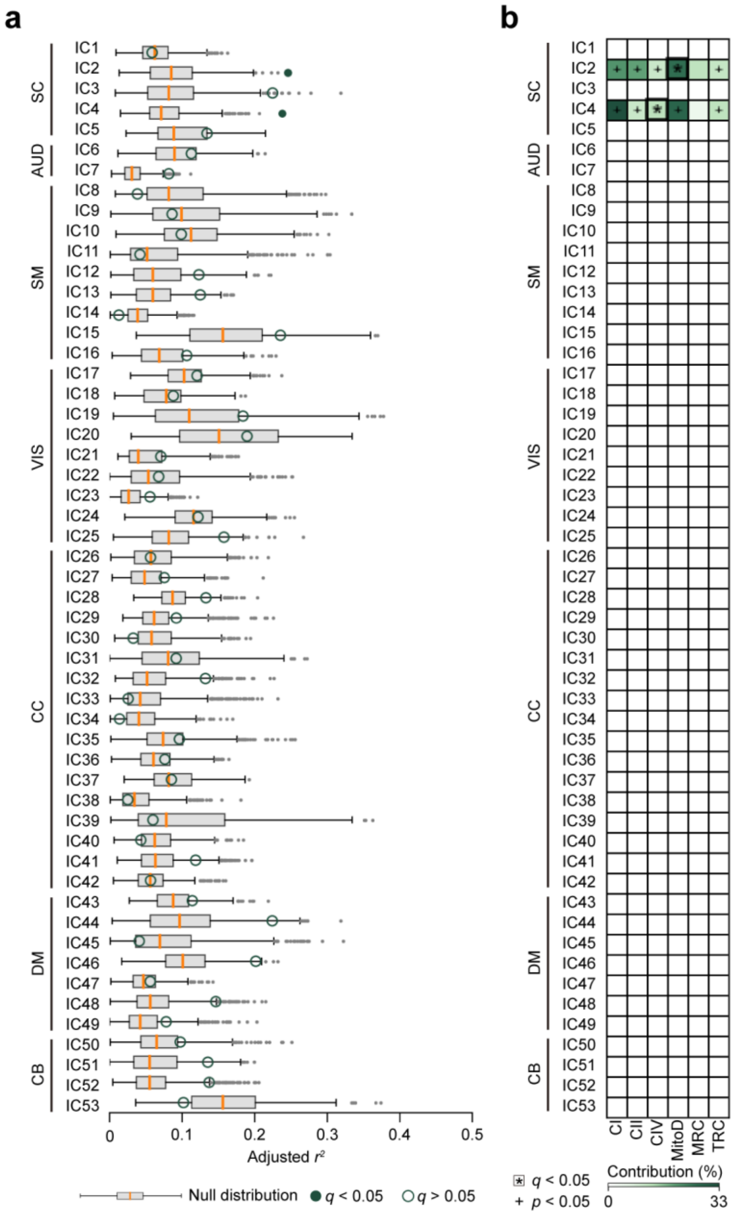

Supplementary Fig. S2. | Mitochondrion associations of ICNs. A multilinear regression framework was used to evaluate the spatial correspondence between 53 ICNs and the distribution of 6 mitochondrial phenotype[3]. Dominance analysis was performed to quantify the unique variance in each ICN map attributable to mitochondrial phenotypes[4]. Statistical significance was assessed using one-sided Moran test (1,000 permutations) to account for spatial autocorrelation[5], with FDR correction applied across 53 adjusted r^2 comparisons and 6×53 relative contribution comparisons. **a** Adjusted r^2 of model fits. Box plots show the median and IQR (25–75%), with whiskers indicating 1.5× IQR from the first or third quartile. **b** Dominance analysis. Source data are provided as a Source Data file.

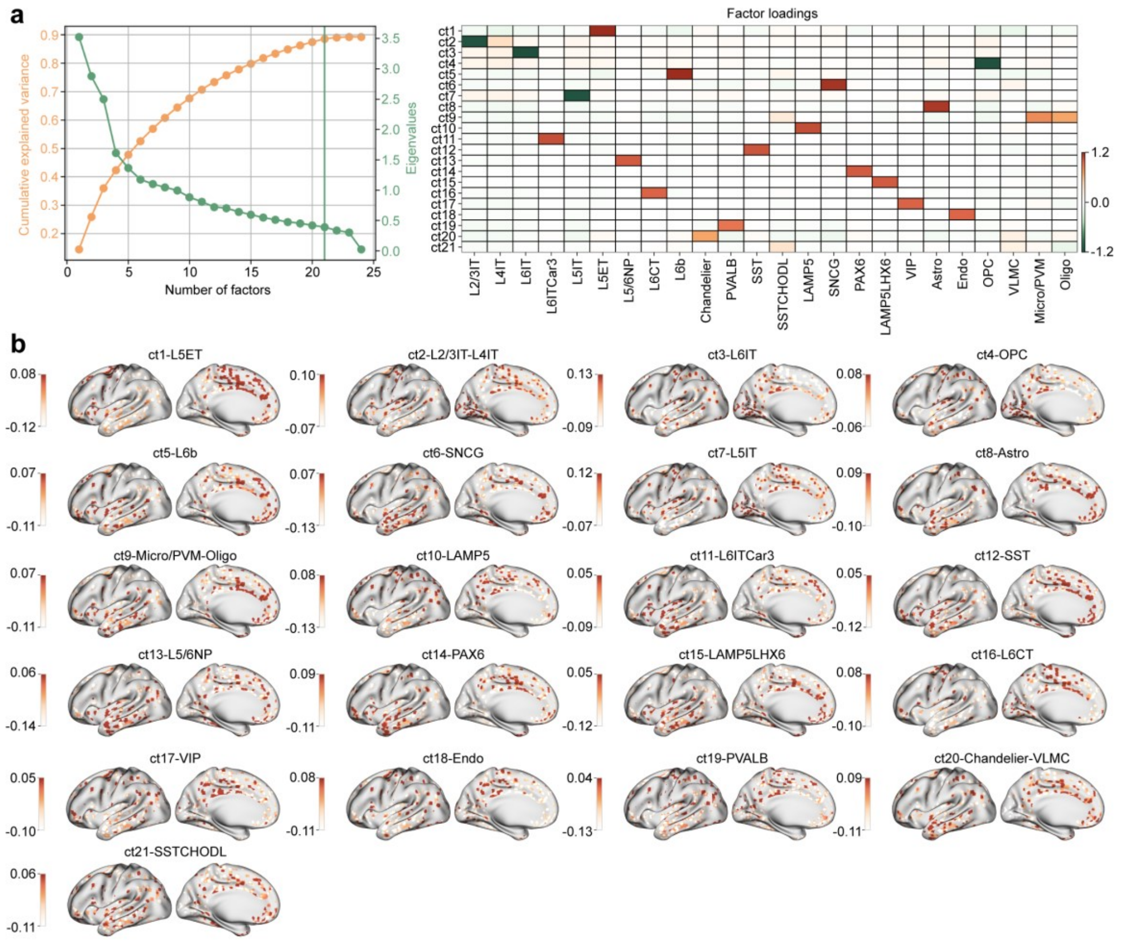

Supplementary Fig. S3. | Factor analysis for cell-type maps. To mitigate collinearity among cell-type predictors while preserving interpretability, we applied factor analysis (minimum residuals, promax oblique rotation)[6]. **a** We retained all unrotated factors explaining ⩾1% of variance (21 factors in total) and named factors by the original atlas with the highest loading. **b** Spatial score maps for each factor. Source data are provided as a Source Data file.

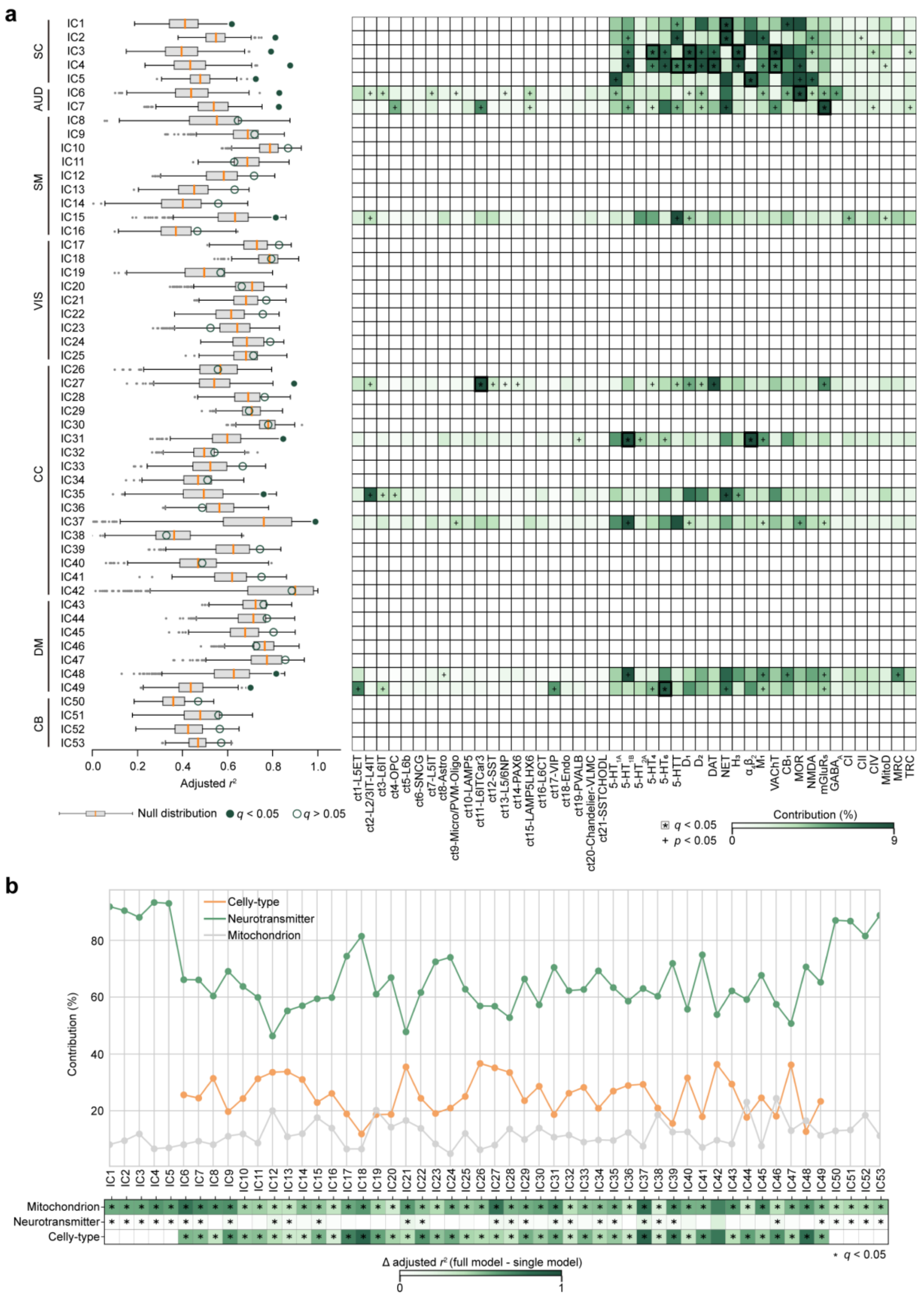

Supplementary Fig. S4. | Synergy and relative contributions of distinct neurobiological scales to ICNs. We fit a multi-scale model (ICN ~ all cell-type, neurotransmitter, and mitochondrion predictors) and single-system models (one system at a time). Model improvement was assessed with F-tests comparing the multi-scale model to each single-scale model, with FDR applied across 3×44

+ 2×9 comparisons. **a** Adjusted r^2 of multi-scale model fits and the relative contribution of each predictor. Boxplots show the median and interquartile range (25–75%), with whiskers indicating 1.5× IQR. **b** Scale-level relative contributions (sum across sub-markers) and model comparison. Source data are provided as a Source Data file.

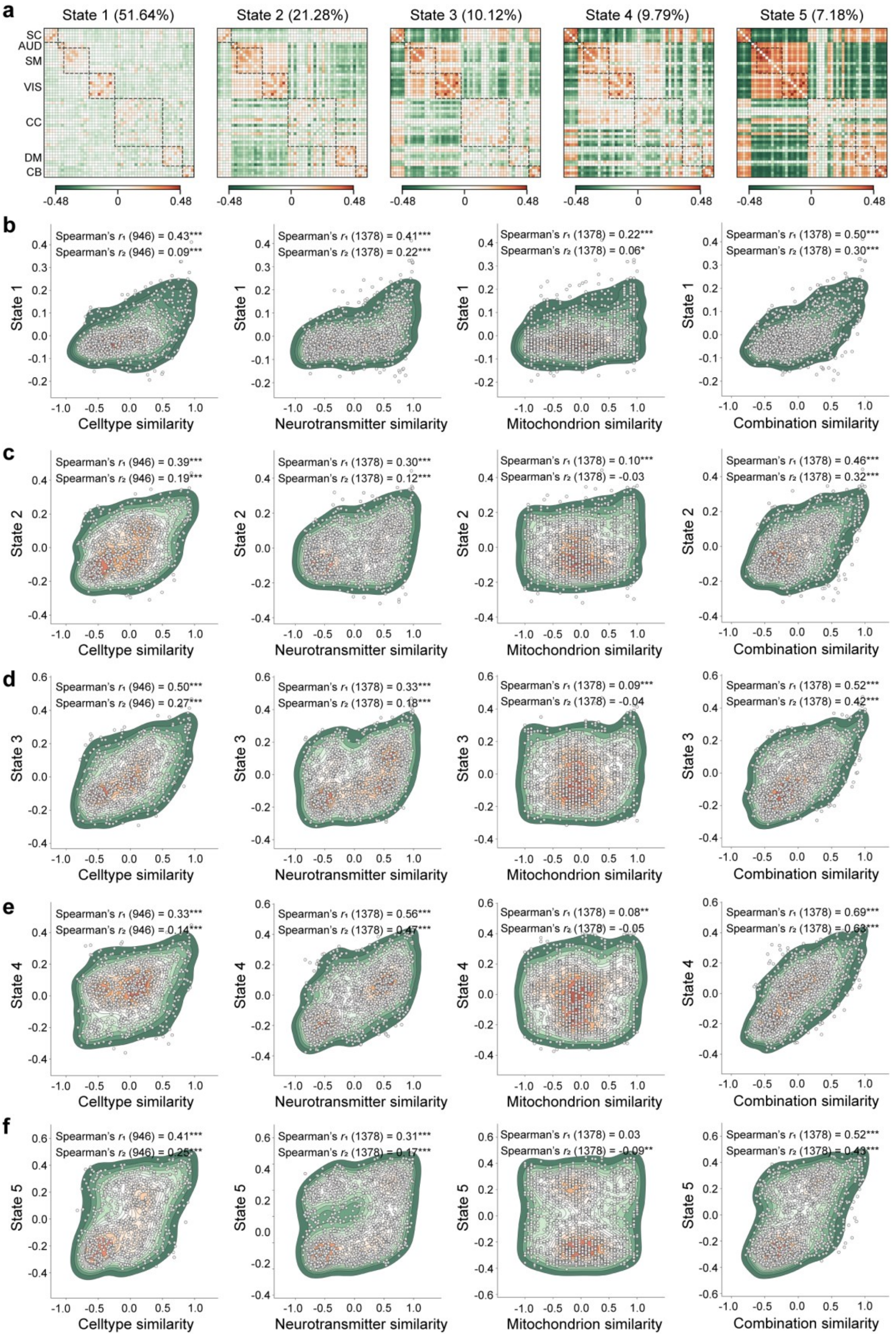

Supplementary Fig. S5. | Cellular and molecular similarity networks relate to dynamic FNCs at the global level. Dynamic FNCs across five states were estimated using a sliding-window

approach[7]. **a** Group-level dynamic FNC for each state. **b-f** Global associations between state-specific dynamic FNC and cellular/molecular similarity networks, assessed using partial Spearman's correlation while controlling for spatial proximity. Note: r_1 denotes the correlation without controlling for spatial proximity; r_2: denotes the correlation after controlling for spatial proximity. *: $p < 0.05$; **: $p < 0.01$; ***: $p < 0.001$. Source data are provided as a Source Data file.

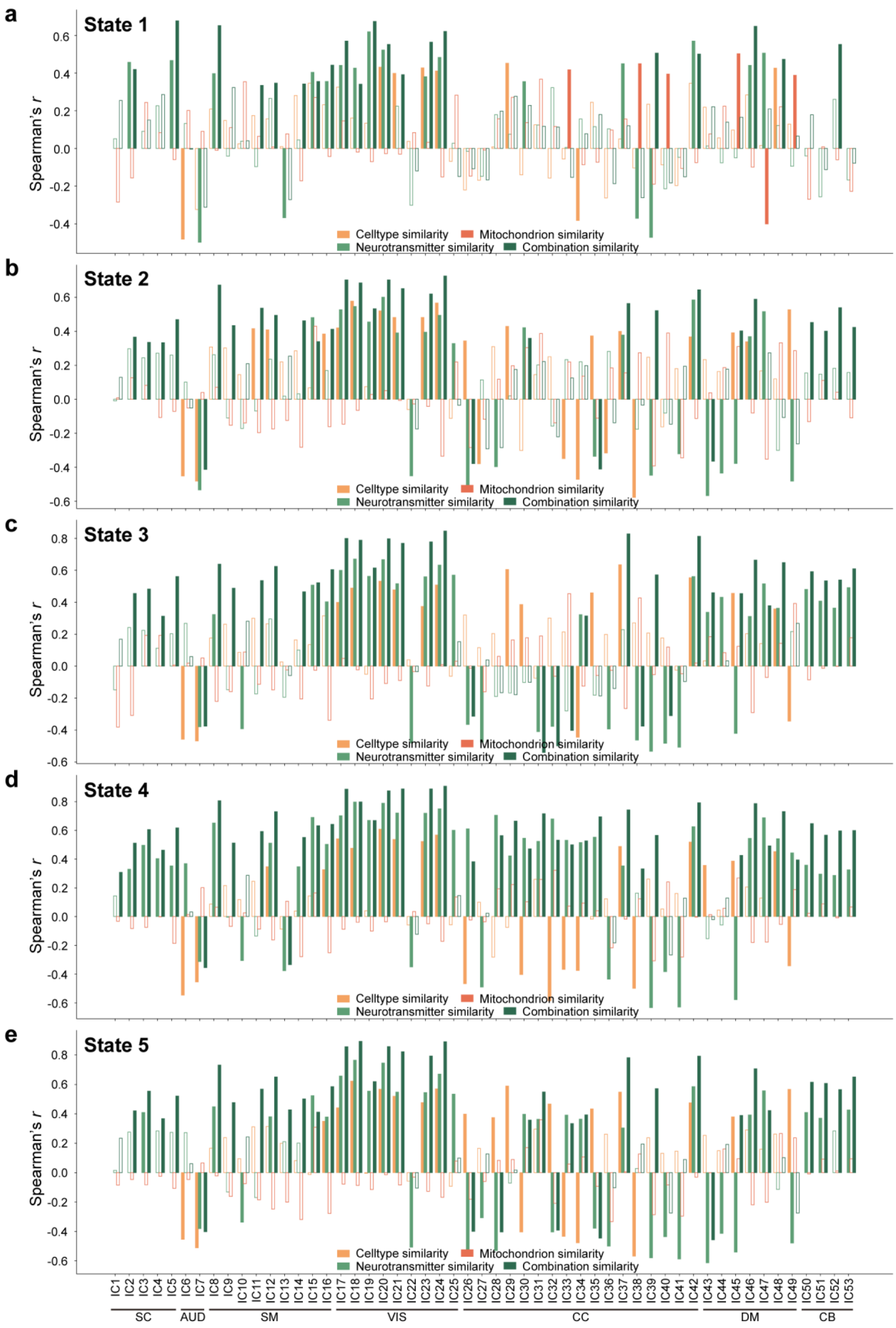

Supplementary Fig. S6. | Cellular and molecular similarity networks relate to dynamic FNCs

at the regional level. a-e Regional associations between state-specific dynamic FNCs and cellular/molecular similarity networks, assessed using partial Spearman's correlation while controlling for spatial proximity. FDR correction was applied across 53 comparisons per modality. In each panel, filled bars indicate statistically significant associations ($q < 0.05$), whereas unfilled bars denote non-significant results. Note: r_1 denotes partial Spearman's correlation without controlling for spatial proximity; r_2: denotes the correlation after controlling for spatial proximity. Source data are provided as a Source Data file.

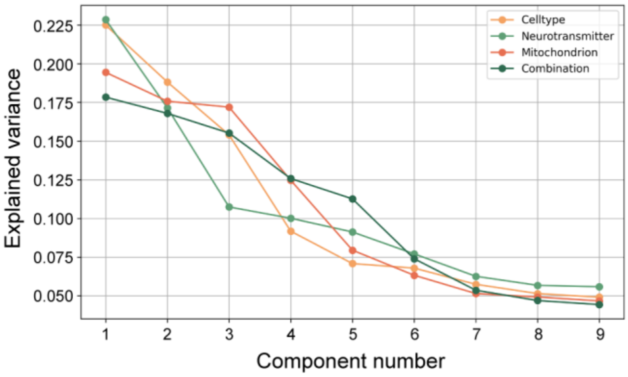

Supplementary Fig. S7. | Explained variance of diffusion embedding. We reduced the dimensionality of the cellular and molecular similarity networks using diffusion embedding[8]. The panel reports explained variance across components. Source data are provided as a Source Data file.

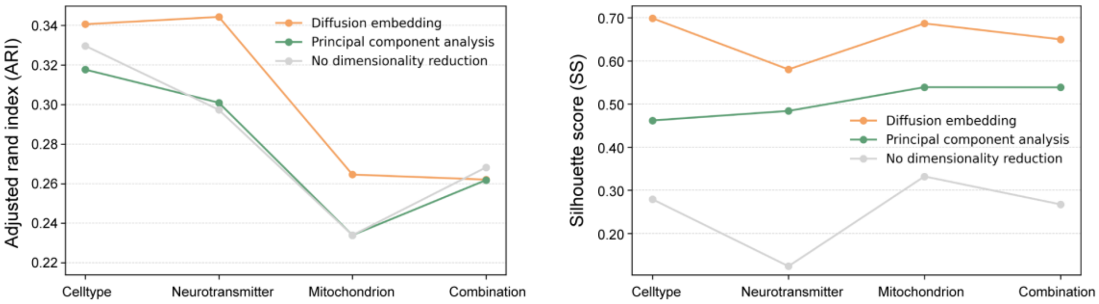

Supplementary Fig. S8. | Impact of dimensionality reduction on clustering. Clustering was performed in three feature spaces: diffusion embedding[8] (first three components), principal component analysis (PCA; first three components), and no dimensionality reduction. Performance was evaluated by the Adjusted Rand Index (ARI), quantifying agreement with functional domains, and the silhouette score (SS), indexing within-cluster cohesion versus between-cluster separation (higher is better for both). Source data are provided as a Source Data file.

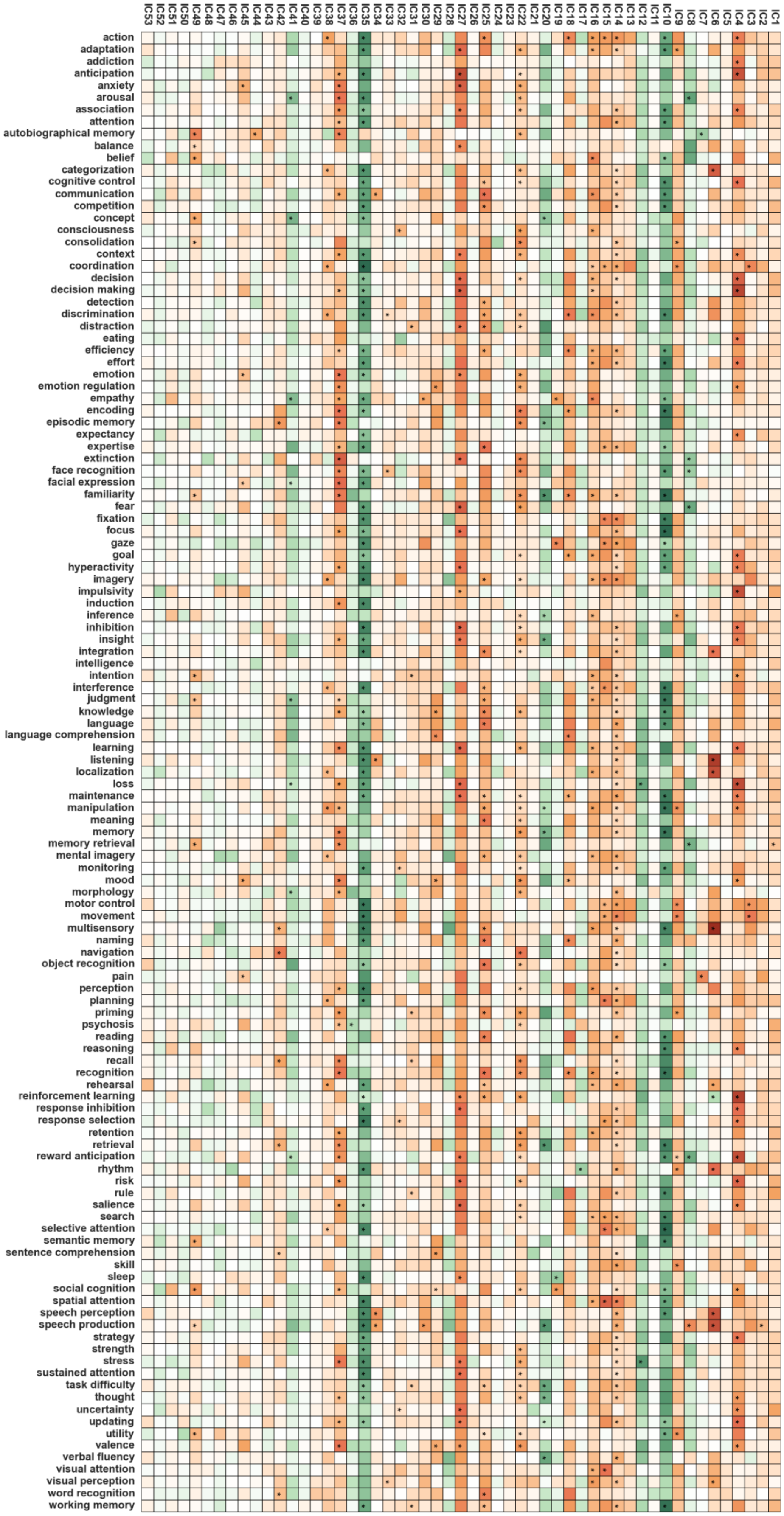

Supplementary Fig. S9. | Cognitive associations of ICNs. The spatial correlations were calculated

between each ICN and 123 cognitive probabilistic measures using Spearman's correlation. The *p*-values were corrected for spatial autocorrelation through 1,000 repetitions of the two-sided Moran test[5], and FDR was used to adjust for 123×53 comparisons per ICN. *: $q < 0.05$. Source data are provided as a Source Data file.

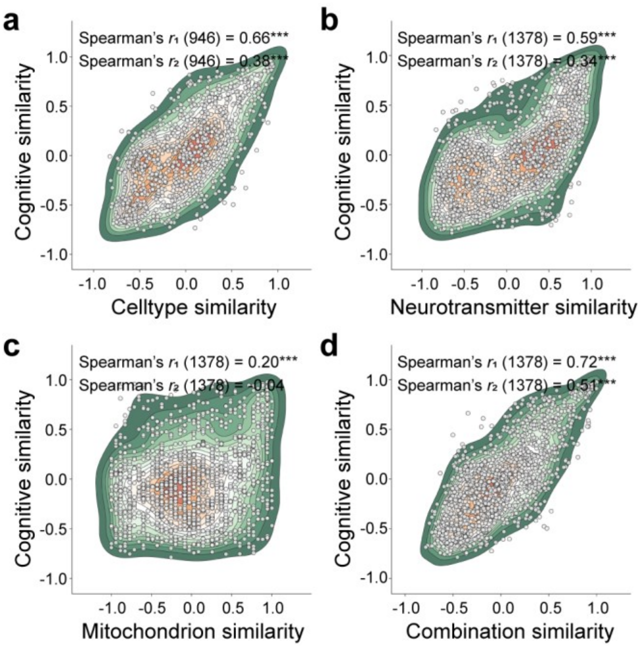

Supplementary Fig. S10. | Cellular and molecular similarity networks relate to cognitive similarity network. Cognitive similarity network was constructed by computing pairwise Spearman correlations between the spatial fingerprints of all ICN pairs. **a-d** Associations between cellular/molecular similarity networks and the cognitive similarity network were evaluated using partial Spearman's correlation, controlling for spatial proximity. Note: r_1 denotes the correlation without controlling for spatial proximity; r_2: denotes the correlation after controlling for spatial proximity. *: $p < 0.05$; **: $p < 0.01$; ***: $p < 0.001$. Source data are provided as a Source Data file.

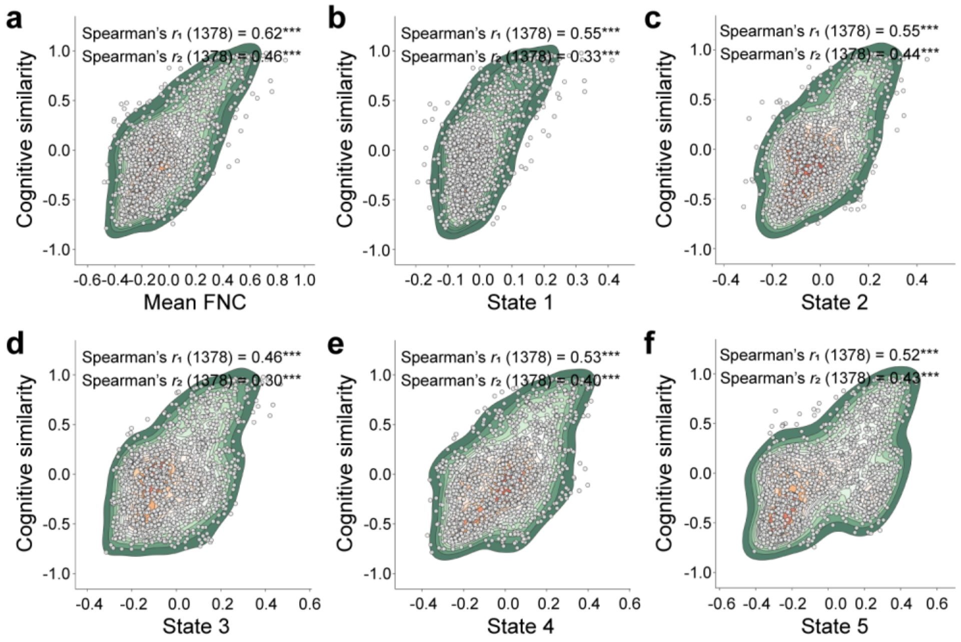

Supplementary Fig. S11. | FNCs relate to cognitive similarity network. a Associations between mean (static) FNC and cognitive similarity network. **b-f** Associations between dynamic FNCs across five distinct connectivity states and cognitive similarity network. All associations were evaluated using partial Spearman's correlation while controlling for spatial proximity. Note: r_1 denotes partial Spearman's correlation without controlling for spatial proximity; r_2: denotes the correlation after controlling for spatial proximity. *: $p < 0.05$; **: $p < 0.01$; ***: $p < 0.001$. Source data are provided as a Source Data file.

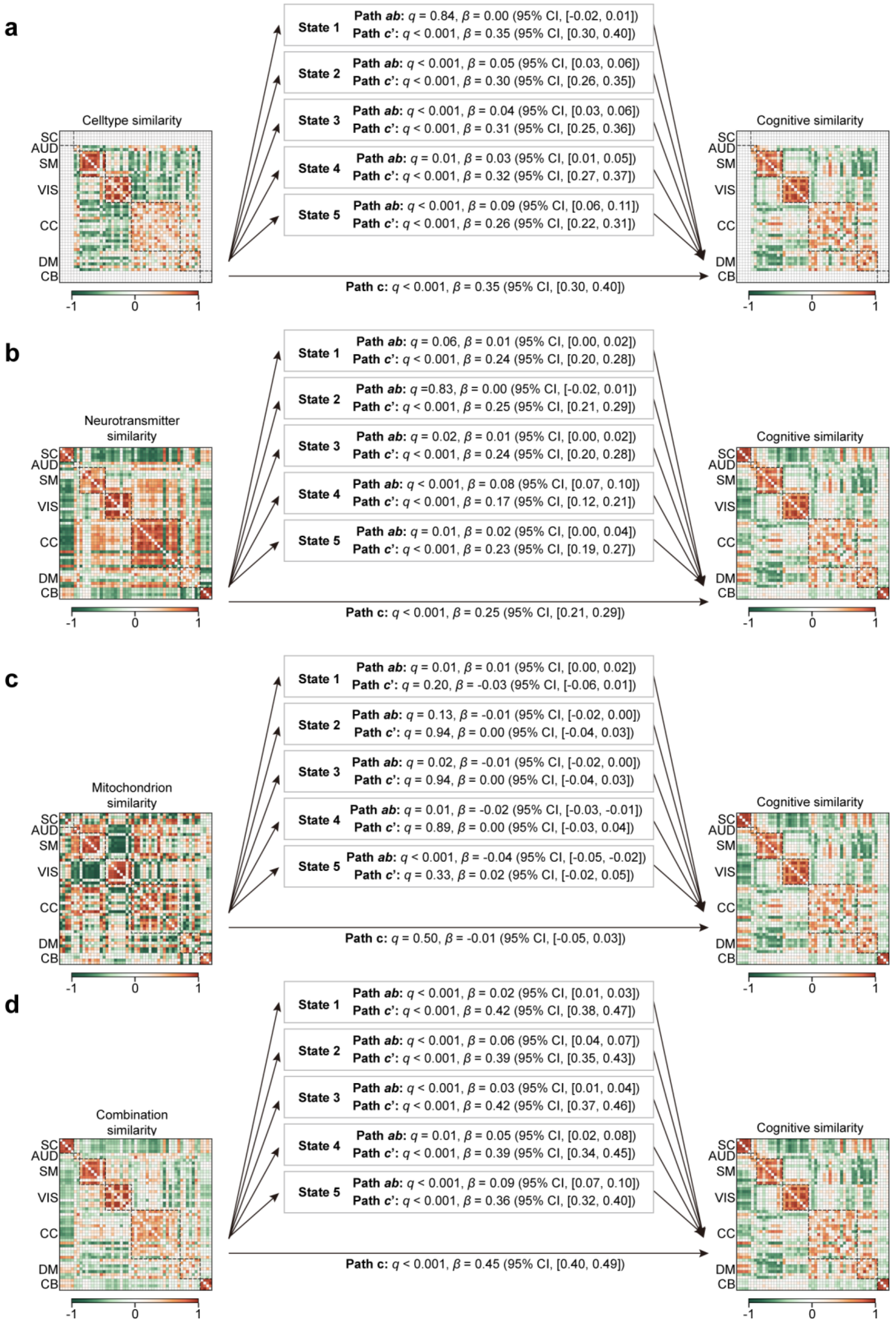

Supplementary Fig. S12. | Dynamic FNCs mediate the connectional associations between cellular and molecular architecture and cognitive function. Mediation analyses at the network level treated cellular or molecular similarity as the predictor, cognitive similarity as the outcome,

and state-specific dynamic FNC as the mediator. Multiple comparisons were controlled using FDR across 5×4 tests. **a** Cell-type; **b** Neurotransmitter; **c** Mitochondrion and **d** Combination. Source data are provided as a Source Data file.

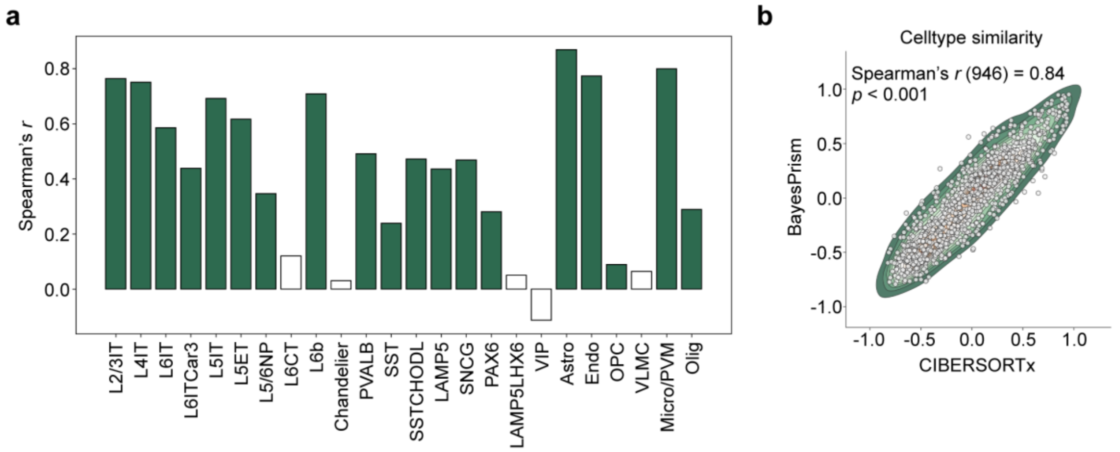

Supplementary Fig. S13. | Agreement across cell-type deconvolution methods. a For each of 24 cell types, cortical spatial maps were estimated with CIBERSORTx[9] and BayesPrism[10]. Cross-method agreement was quantified by Spearman correlation per cell-type (filled bars = FDR $q < 0.05$ across 24 tests). **b** Agreement between the cell-type similarity networks derived from the two methods, summarized by the Spearman correlation of corresponding edge weights. Source data are provided as a Source Data file.

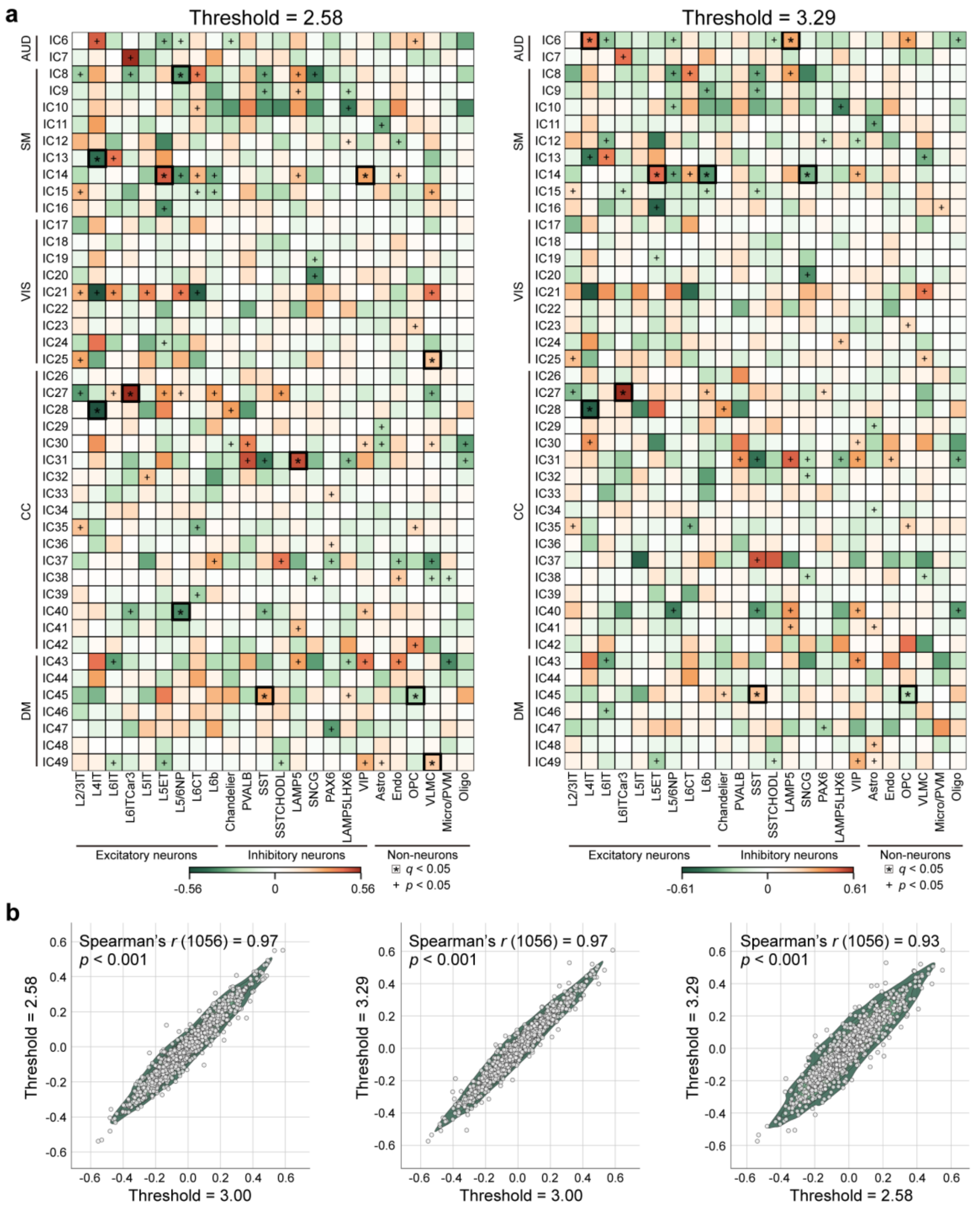

Supplementary Fig. S14. | Consistency of ICN–cell-type associations across thresholds. a Association results at different thresholds (2.58 [two-sided $p = 0.01$] and 3.29 [two-sided $p = 0.001$]. **b** Between-threshold similarity of results. Source data are provided as a Source Data file.

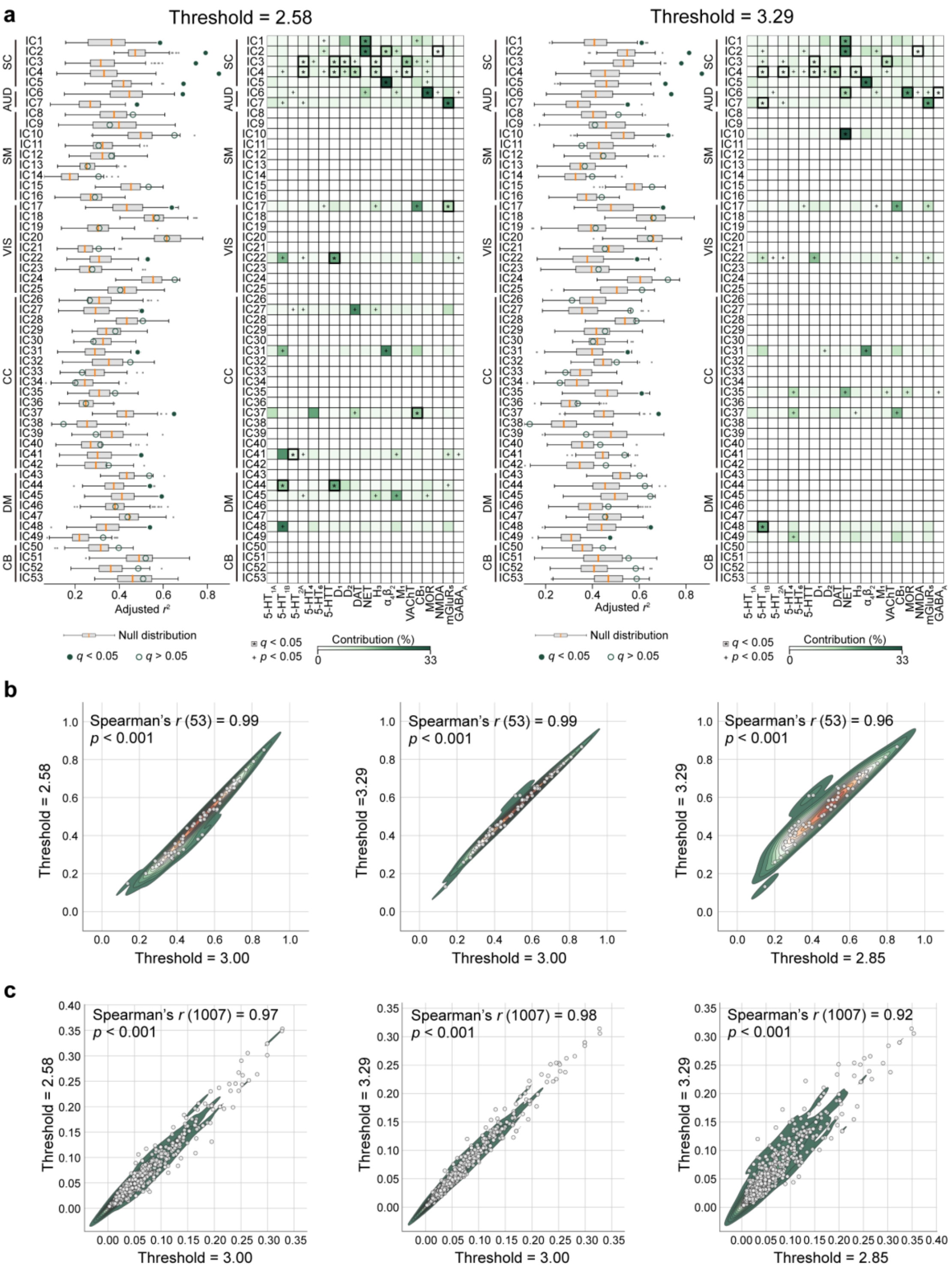

Supplementary Fig. S15. | Consistency of ICN–neurotransmitter associations across thresholds. **a** Association results at different thresholds (2.58 [two-sided $p = 0.01$] and 3.29 [two-sided $p = 0.001$]. **b** Between-threshold similarity of adjusted r^2. **c** Between-threshold similarity of contribution. Source data are provided as a Source Data file.

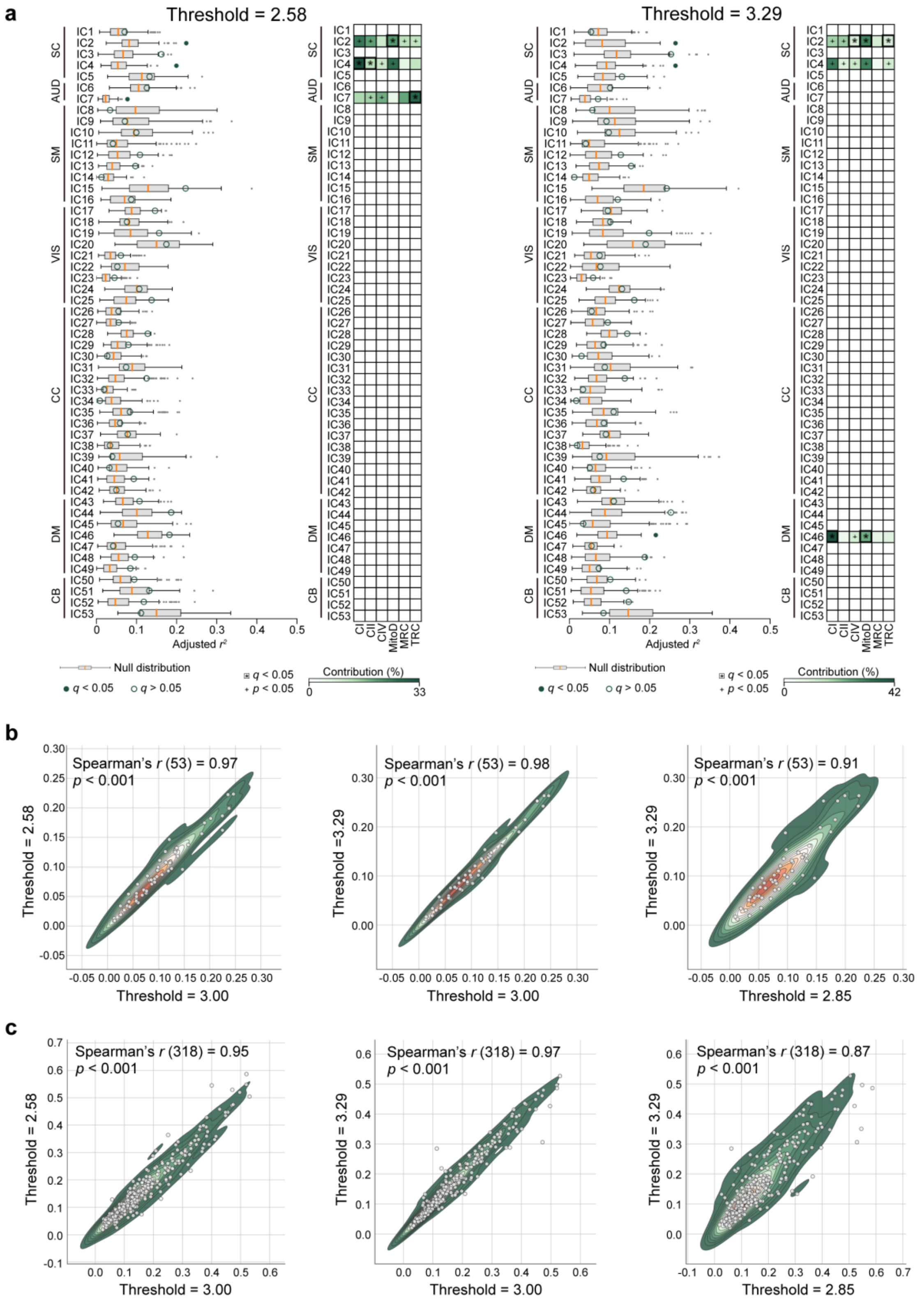

Supplementary Fig. S16. | Consistency of ICN–mitochondrion associations across thresholds. **a** Association results at different thresholds (2.58 [two-sided $p = 0.01$] and 3.29 [two-sided $p = 0.001$]. **b** Between-threshold similarity of adjusted r^2. **c** Between-threshold similarity of contribution. Source data are provided as a Source Data file.

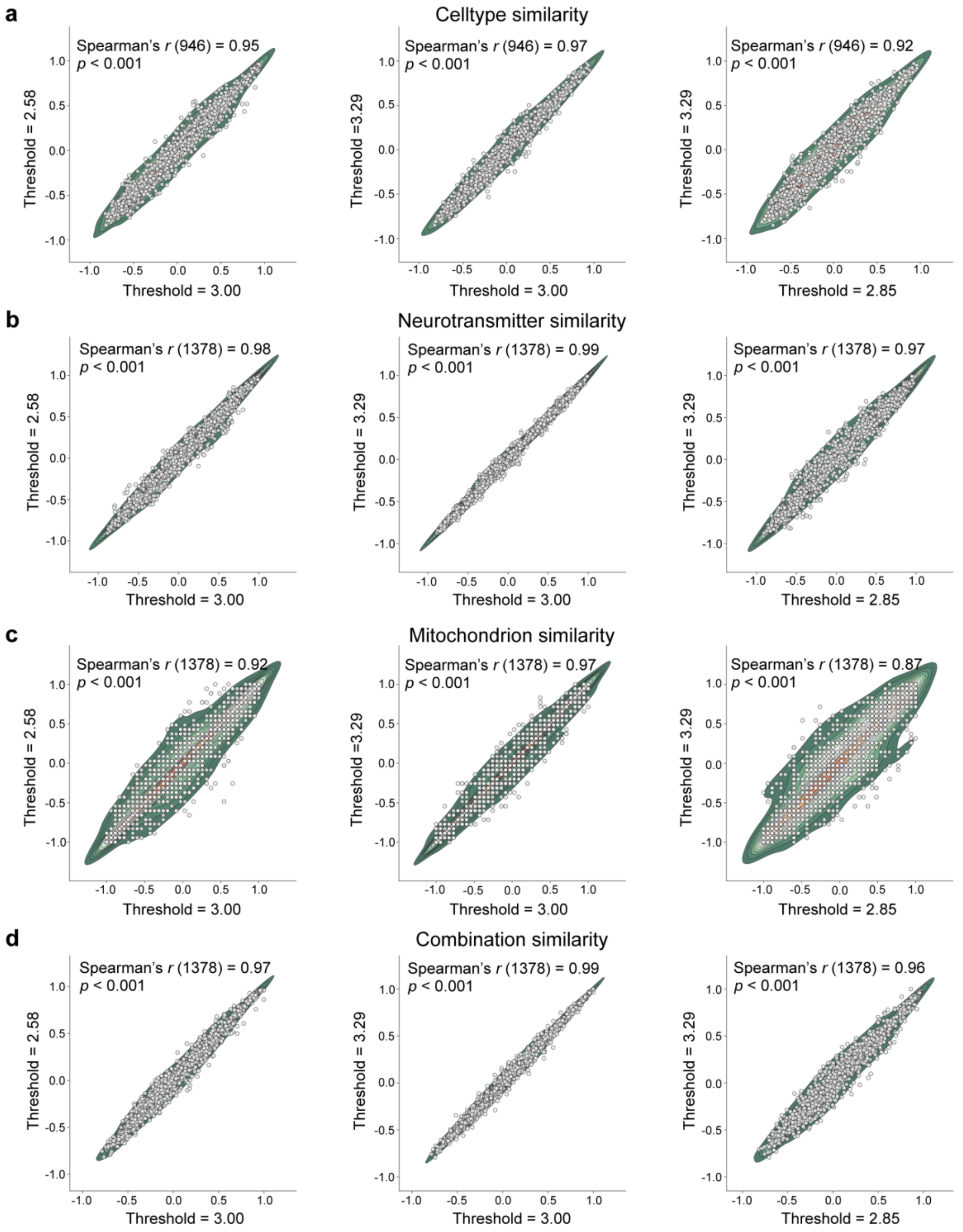

Supplementary Fig. S17. | Consistency of cell-type/molecular similarity networks across thresholds. a Cell-type similarity. **b** Neurotransmitter similarity. **c** Mitochondrion similarity. **d** Combination similarity. Source data are provided as a Source Data file.

References

1. Du, Y., *et al.* NeuroMark: An automated and adaptive ICA based pipeline to identify reproducible fMRI markers of brain disorders. *Neuroimage Clin* **28**, 102375 (2020).
2. Correa, N.M., Li, Y.O., Adalı, T. & Calhoun, V.D. Canonical Correlation Analysis for Feature-Based Fusion of Biomedical Imaging Modalities and Its Application to Detection of Associative Networks in Schizophrenia. *IEEE J Sel Top Signal Process* **2**, 998-1007 (2008).
3. Mosharov, E.V., *et al.* A human brain map of mitochondrial respiratory capacity and diversity. *Nature* (2025).
4. Azen, R. & Budescu, D.V. The dominance analysis approach for comparing predictors in multiple regression. *Psychol Methods* **8**, 129-148 (2003).
5. Vos de Wael, R., *et al.* BrainSpace: a toolbox for the analysis of macroscale gradients in neuroimaging and connectomics datasets. *Commun Biol* **3**, 103 (2020).
6. Lotter, L.D., *et al.* Regional patterns of human cortex development correlate with underlying neurobiology. *Nat Commun* **15**, 7987 (2024).
7. Fu, Z., Sui, J., Iraji, A., Liu, J. & Calhoun, V.D. Cognitive and psychiatric relevance of dynamic functional connectivity states in a large (N > 10,000) children population. *Molecular Psychiatry* **30**, 402-413 (2024).
8. Margulies, D.S., *et al.* Situating the default-mode network along a principal gradient of macroscale cortical organization. *Proc Natl Acad Sci U S A* **113**, 12574-12579 (2016).
9. Newman, A.M., *et al.* Determining cell type abundance and expression from bulk tissues with digital cytometry. *Nature biotechnology* **37**, 773-782 (2019).
10. Chu, T., Wang, Z., Pe'er, D. & Danko, C.G. Cell type and gene expression deconvolution with BayesPrism enables Bayesian integrative analysis across bulk and single-cell RNA sequencing in oncology. *Nat Cancer* **3**, 505-517 (2022).
