# Supplementary material for: Cellular and molecular associations with intrinsic brain organization
Source: Nat Commun. 2025 Nov 26;16:11641. doi: 10.1038/s41467-025-66291-w (PMC12748960; doi:10.1038/s41467-025-66291-w)
Supplement: Supplementary file 2 — Reporting Summary [file 41467_2025_66291_MOESM2_ESM.pdf]

nature portfolio

Corresponding author(s): Vince D. Calhoun

Last updated by author(s): Oct 22, 2025

Reporting Summary

Nature Portfolio wishes to improve the reproducibility of the work that we publish. This form provides structure for consistency and transparency in reporting. For further information on Nature Portfolio policies, see our Editorial Policies and the Editorial Policy Checklist.

Statistics

For all statistical analyses, confirm that the following items are present in the figure legend, table legend, main text, or Methods section.

n/a	Confirmed	
☐	☒	The exact sample size (*n*) for each experimental group/condition, given as a discrete number and unit of measurement
☐	☒	A statement on whether measurements were taken from distinct samples or whether the same sample was measured repeatedly
☐	☒	The statistical test(s) used AND whether they are one- or two-sided<br>*Only common tests should be described solely by name; describe more complex techniques in the Methods section.*
☐	☒	A description of all covariates tested
☐	☒	A description of any assumptions or corrections, such as tests of normality and adjustment for multiple comparisons
☐	☒	A full description of the statistical parameters including central tendency (e.g. means) or other basic estimates (e.g. regression coefficient) AND variation (e.g. standard deviation) or associated estimates of uncertainty (e.g. confidence intervals)
☐	☒	For null hypothesis testing, the test statistic (e.g. *F*, *t*, *r*) with confidence intervals, effect sizes, degrees of freedom and *P* value noted<br>*Give P values as exact values whenever suitable.*
☒	☐	For Bayesian analysis, information on the choice of priors and Markov chain Monte Carlo settings
☒	☐	For hierarchical and complex designs, identification of the appropriate level for tests and full reporting of outcomes
☐	☒	Estimates of effect sizes (e.g. Cohen's *d*, Pearson's *r*), indicating how they were calculated

Our web collection on statistics for biologists contains articles on many of the points above.

Software and code

Policy information about availability of computer code

Data collection	No software was used in the data collection process.
Data analysis	Custom data and code for our analysis is accessible via the following links: https://trendscenter.org/data/ and https://github.com/FelixFengCN/ICNs-annotation.<br>MATLAB R2020b, Python 3.9.21, and R 4.1 scripts were used for the following major analysis:<br>Intrinsic connectivity networks (ICNs) and functional network connectivity (FNC) via SPM12 (http://www.fil.ion.ucl.ac.uk/spm/) and GIFT toolbox (http://trendscenter.org/software/gift)<br>AHBA sample preprocessing via abagen toolbox (https://abagen.readthedocs.io/en/stable)<br>Cell-type imputation based on single-nucleus droplet-based sequencing (snDrop-seq) data via CIBERSORTx (https://cibersortx.stanford.edu/)<br>Relative importance analysis via relaimpo R package (https://cran.r-project.org/web/packages/relaimpo/index.html)<br>Moran test and diffusion embedding method via BrainSpace (https://brainspace.readthedocs.io/en/latest/index.html)<br>Mediation analyses using PROCESS R package (https://search.r-project.org/CRAN/refmans/bruceR/html/PROCESS.html).

For manuscripts utilizing custom algorithms or software that are central to the research but not yet described in published literature, software must be made available to editors and reviewers. We strongly encourage code deposition in a community repository (e.g. GitHub). See the Nature Portfolio guidelines for submitting code & software for further information.

Data

Policy information about availability of data

All manuscripts must include a data availability statement. This statement should provide the following information, where applicable:

- Accession codes, unique identifiers, or web links for publicly available datasets
- A description of any restrictions on data availability
- For clinical datasets or third party data, please ensure that the statement adheres to our policy

rs-fMRI data were downloaded from http://www.humanconnectomeproject.org/data.
Neuromark_fMRI_1.0 template was available at http://trendscenter.org/software/gifti.
Human microarray gene expression data obtained from bulk samples of 6 postmortem brains were downloaded from AHBA dataset (http://human.brain-map.org/).
snDrop-seq data obtained by Jorstad et al. were downloaded from cellxgene-census (https://cellxgene.cziscience.com/collections/d17249d2-0e6e-4500-abb8-e6c93fa1ac6f).
Neurotransmitter PET maps were downloaded at https://github.com/netneurolab/hansen_receptors.
Mitochondrial phenotype maps were downloaded at https://neurovault.org/collections/16418/.
Cognitive probabilistic maps were downloaded from Neurosynth (https://github.com/neurosynth/neurosynth).
Derived data generated in this study have been deposited at https://trendscenter.org/data/ and https://github.com/FelixFengCN/ICNs-annotation.

Research involving human participants, their data, or biological material

Policy information about studies with human participants or human data. See also policy information about sex, gender (identity/presentation), and sexual orientation and race, ethnicity and racism.

Reporting on sex and gender	HCP include 356 male and 467 female (in gender).<br>AHBA include 6 donors, 5 were male and 1 was female (in sex).<br>snRNA-seq data from Jorstad et al. has 4 males and 2 females.
Reporting on race, ethnicity, or other socially relevant groupings	Grouping was not relevant to our analyses because they were always conducted on the full set of available data.
Population characteristics	HCP mean age 28.8 years, range 22-37 years.<br>AHBA mean age 42.5 years, range 24-57 years.<br>snRNA-seq from Jorstad et al. mean age 45.7, range 29-60.
Recruitment	Anonymized open access data were used for the present analysis.<br>Recruitment details of HCP can be seen at: Van Essen, D.C., Smith, S.M., Barch, D.M., et al. (2013). The WU-Minn Human Connectome Project: An overview. NeuroImage 80, 62–79. 10.1016/j.neuroimage.2013.05.041.<br>AHBA: Hawrylycz, M.J., Lein, E.S., Guillozet-Bongaarts, A.L., et al. (2012). An anatomically comprehensive atlas of the adult human brain transcriptome. Nature 489, 391–399. 10.1038/nature11405.<br>snRNA-seq provided by Jorstad et al.: Jorstad, N. L., Close, J., Johansen, N., et al. (2023). Transcriptomic cytoarchitecture reveals principles of human neocortex organization. Science, 382(6667), eadf6812.
Ethics oversight	Not applicable to the current study, where anonymized open access data were used.

Note that full information on the approval of the study protocol must also be provided in the manuscript.

Field-specific reporting

Please select the one below that is the best fit for your research. If you are not sure, read the appropriate sections before making your selection.

☒ Life sciences ☐ Behavioural & social sciences ☐ Ecological, evolutionary & environmental sciences

For a reference copy of the document with all sections, see nature.com/documents/nr-reporting-summary-flat.pdf

Life sciences study design

All studies must disclose on these points even when the disclosure is negative.

Sample size	Our analyses utilized open-access genetic and neuroimaging consortia datasets, including all donors from the Allen Human Brain Atlas (AHBA) and Jorstad single-nucleus RNA-seq dataset, and resting-state functional connectivity data from 823 participants of the Human Connectome Project (HCP). As the study was based on publicly available large-scale datasets, no a priori sample size calculations were performed. The sample sizes are consistent with or exceed those used in prior studies of similar scope, providing sufficient power for the reported analyses.
Data exclusions	A rigorous criterion was implemented for subject selection to ensure high-quality data. For fMRI, we selected data with the properties: (1) data with head motions less than 3º rotations and 3 mm transitions along the whole scanning period; (2) data with more than 120 time points in fMRI acquisition; (3) data providing a successful normalization in the full brain. In terms of the third point, whether fMRI data have good normalization to the template is important in multi-subject ICA. We evaluated the normalization quality of data by comparing the subject-specific mask and the group mask. This method was applied to each study's fMRI data separately. First, using the volume in the first time

point, we calculated the individual mask for each subject by setting voxels showing greater values than 90% of the whole brain mean to 1. Next, we yielded a group mask by setting voxels included in more than 90% of the individual masks to 1. Then, for each subject, we calculated the correlations between the group mask and the individual mask. The correlations were calculated using voxels within the top 10 slices of the mask, within the bottom 10 slices of the mask, and within the whole mask, resulting in three correlation values for each subject. If a subject had correlations larger than the specified thresholds, we included this subject for further fMRI analysis. After the quality control of the data processing, 179 subjects in HCP were excluded.

Replication

We used intrinsic connectivity networks (ICNs) derived from the Neuromark_fMRI_1.0 template, which has been demonstrated to be robust and reproducible across multiple independent datasets, including GSP and HCP. The cell-type analysis was based on abundance maps imputed from the Allen Human Brain Atlas (AHBA) and the Jorstad single-nucleus RNA-seq dataset, which represent the most comprehensive and reproducible datasets currently available for the human cortex. The neurotransmitter receptor atlas and cognitive probabilistic maps were obtained from large multi-study consortia and are considered highly reliable. For mitochondrial phenotypes, we used the unique high–spatial-resolution atlas derived from single-nucleus RNA data. All analyses were performed using standardized, publicly available datasets and transparent workflows, ensuring full computational reproducibility.

Randomization

Randomization was not relevant to our analyses because they were always conducted on the full set of available data and did not include "case/control" experimental design.

Blinding

Blinding was not relevant to our study. Our study did not distinguish or define experimental groups.

Reporting for specific materials, systems and methods

We require information from authors about some types of materials, experimental systems and methods used in many studies. Here, indicate whether each material, system or method listed is relevant to your study. If you are not sure if a list item applies to your research, read the appropriate section before selecting a response.

Materials & experimental systems

n/a	Involved in the study
☒	☐ Antibodies
☒	☐ Eukaryotic cell lines
☒	☐ Palaeontology and archaeology
☒	☐ Animals and other organisms
☒	☐ Clinical data
☒	☐ Dual use research of concern
☒	☐ Plants

Methods

n/a	Involved in the study
☒	☐ ChIP-seq
☒	☐ Flow cytometry
☐	☒ MRI-based neuroimaging

Plants

Seed stocks

Report on the source of all seed stocks or other plant material used. If applicable, state the seed stock centre and catalogue number. If plant specimens were collected from the field, describe the collection location, date and sampling procedures.

Novel plant genotypes

Describe the methods by which all novel plant genotypes were produced. This includes those generated by transgenic approaches, gene editing, chemical/radiation-based mutagenesis and hybridization. For transgenic lines, describe the transformation method, the number of independent lines analyzed and the generation upon which experiments were performed. For gene-edited lines, describe the editor used, the endogenous sequence targeted for editing, the targeting guide RNA sequence (if applicable) and how the editor was applied.

Authentication

Describe any authentication procedures for each seed stock used or novel genotype generated. Describe any experiments used to assess the effect of a mutation and, where applicable, how potential secondary effects (e.g. second site T-DNA insertions, mosiacism, off-target gene editing) were examined.

Magnetic resonance imaging

Experimental design

Design type

Resting state functional magnetic resonance imaging

Design specifications

Four 15-minute rest-run per subject

Behavioral performance measures

No individual behavioral performance measures were used in this study

Acquisition

Imaging type(s)	Resting state functional magnetic resonance imaging
Field strength	3.0 T
Sequence & imaging parameters	TR=720ms, TE=33.1ms, spatial resolution=2*2*2mm^3, more details can be found at db.humanconnectome.org
Area of acquisition	Whole brain

Diffusion MRI ☐ Used ☒ Not used

Preprocessing

Preprocessing software	We used preprocessed rs-fMRI data from http://www.humanconnectomeproject.org/data.<br>ICNs and FNC via SPM12 (http://www.fil.ion.ucl.ac.uk/spm/) and GIFT toolbox (http://trendscenter.org/software/gift).
Normalization	The normalization was based on the standard HCP preprocessing, and the subsequent steps were based on the standard GIFT processing (http://trendscenter.org/software/gift).
Normalization template	The normalization was based on the standard HCP preprocessing, and the subsequent steps were based on the standard GIFT processing (http://trendscenter.org/software/gift).
Noise and artifact removal	After HCP pre-processed data were downloaded, to further minimize residual noise in the ICN TCs, four additional postprocessing steps were applied: (1) detrending to remove linear, quadratic, and cubic trends; (2) outlier detection and removal; (3) multiple regression of six head motion parameters (three translations and three rotations) along with their temporal derivatives; and (4) band-pass filtering (0.01–0.15 Hz).
Volume censoring	The standard HCP preprocessing.

Statistical modeling & inference

Model type and settings	Spearman correlation, Moran test, multivariate linear regression models analysis, mediation analysis
Effect(s) tested	We tested whether spatial and connectional correlations between ICNs and cellular/molecular maps and whether the functional network mediates the relationship between cellular/molecular maps and cognitive maps.

Specify type of analysis: ☐ Whole brain ☒ ROI-based ☐ Both

Anatomical location(s)	ICN template was from the Neuromark_fMRI_1.0:<br>Du, Y., et al. NeuroMark: An automated and adaptive ICA based pipeline to identify reproducible fMRI markers of brain disorders. Neuroimage Clin 28, 102375 (2020).<br>AHBA voxel location:<br>Hawrylycz, M.J., et al. An anatomically comprehensive atlas of the adult human brain transcriptome. Nature 489, 391-399 (2012).
Statistic type for inference<br>(See Eklund et al. 2016)	Analysis were conducted at independent component parcel level and network level.
Correction	FDR was for multiple comparisons.

Models & analysis

n/a	Involved in the study
☐	☒ Functional and/or effective connectivity
☐	☒ Graph analysis
☐	☒ Multivariate modeling or predictive analysis

Functional and/or effective connectivity	Pearson's correlation were computed between the denoised ICN time courses to generate individual-level FNC matrices.
Graph analysis	Analysis were conducted at connectional correlations between FNC and cellular/molecular similarity networks.
Multivariate modeling and predictive analysis	Multivariate linear regression models analysis with Moran test was used to calculate the spatial correlation between each ICNs and 19 neurotransmitter maps or 6 mitochondrial phenotype maps.
